# Robotic Surgery for Benign Hysterectomy: A Real-World Study From India

**DOI:** 10.7759/cureus.74932

**Published:** 2024-12-01

**Authors:** Raman Patel, Reitu Patel

**Affiliations:** 1 Department of Gynecology and Obstetrics, Zydus Hospitals, Ahmedabad, IND

**Keywords:** adnexal masses, endometrioma, hysterectomy, robotic surgery, uterine leiomyoma

## Abstract

Background

In gynecology, hysterectomy is a common surgical procedure for benign conditions. This study was conducted to assess the short-term clinical outcomes of robotic-assisted hysterectomy in the Indian population.

Methods

We performed a retrospective chart review of patients who underwent robotic-assisted benign hysterectomy procedures between December 2021 and July 2024. A single senior surgeon collected clinical data points related to patient demographics, comorbidities, and surgical outcomes.

Results

A total of 113 patients with a mean age of 45.39 ± 9.04 years and a body mass index of 29.07 ± 4.69 kg/m^2^ were included in the study. The mean operating room time was 178.41 ± 18.65 minutes, while the estimated blood loss and length of hospital stay were 23.85 ± 5.84 mL and 2.86 ± 0.42 days, respectively. The mean ambulation time was 21.22 ± 3.10 hours. There were only three post-operative complications: urine leakage in two (1.77%) patients and intestinal obstruction due to an ileal adhesion in one (0.88%) patient.

Conclusion

There are potential advantages to the robotic-assisted hysterectomy in terms of blood loss, length of hospital stay, and intra- and post-operative complications. The study's conclusions support the use of robotic assistance in the surgical management of benign hysterectomy.

## Introduction

Hysterectomy for benign pathologies is a common surgical procedure in gynecology. A multi-institutional study from India involving 764 cases reported that uterine leiomyoma (31%), endometrioma (8%), pelvic endometriosis (6%), adenomyosis (4%), vault prolapse (3%), adnexal masses (1%), abnormal uterine bleeding (1%), and ectopic pregnancy (1%) are among the most common benign conditions requiring surgical intervention [1]. It is well-documented that millions of hysterectomies are carried out annually worldwide [2]. The percentage of patients with hysterectomy in the reproductive years (18-44 years) in the USA has reached about 18%, while those in the remaining age groups have reached around 48% [3]. In addition, in India, women who are 30 to 49 years old have a high rate of hysterectomy, at roughly 6% [4]. The literature indicates that hysterectomy for benign gynecological diseases can be performed using five major approaches: abdominal hysterectomy (AH), vaginal hysterectomy (VH), laparoscopic hysterectomy (LH), robotic-assisted hysterectomy (RH), and vaginal natural orifice transluminal endoscopic surgery (V-NOTES) [5,6]. Moreover, several factors had a major impact on the choice of surgery, including the size and shape of the uterus, the degree of extrauterine disease, pelvic adhesions, mobility, surgeon training, cost-effectiveness, and available resources [6,7].

Laparoscopic and robotic-assisted VH are currently classified as minimally invasive surgeries (MIS) [6]. The American College of Obstetricians and Gynecologists (ACOG) recommends using vaginal and laparoscopic procedures as the MIS methods for hysterectomy [8]. MIS has several advantages for the patient, including less trauma, a shorter surgical time, decreased intra-operative blood loss, decreased intra- and post-operative complications, higher patient satisfaction, and less morbidity [9-13]. In 2005, the FDA approved the Da Vinci Surgical System by Intuitive Surgical Inc., Sunnyvale, California, USA, for gynecologic surgery [10]. A multi-institutional study from India showed how quickly robotic approaches were accepted for gynecological procedures, with the number of cases operated between 2016 and 2021 just doubling as compared to the period between 2011 and 2015 (67.4% vs. 32.6%) [1]. In addition, a comprehensive analysis of RH, VH, AH, and LH by high-volume surgeons concluded that the robotic technique had superior outcomes in terms of intra-operative and post-operative complications [14]. However, inconsistent findings have been observed in meta-analyses of surgical outcomes for RH versus LH. While some studies on benign hysterectomy have demonstrated evidence of improved outcomes using robotic techniques (less blood loss, shorter operative time, shorter length of hospital stay, better cosmesis, and decreased rates of conversion and complication), others have not confirmed the superiority of robotic surgery over laparoscopic surgery [13,15-17]. Also, robotic-assisted surgery has certain ethical concerns. The adequacy of the consent process may be an ethical dilemma due to patients not fully understanding the limitations or risks associated with robotic-assisted surgery. Additionally, the cost of robotic systems restricts their availability to well-funded hospitals, resulting in disparities in access based on socioeconomic or geographical factors. Furthermore, the techniques used for hysterectomy have significantly changed over time [18]. Open AH was the preferred method for 51.4% of all hysterectomies in 2002, but it has decreased to 1.4% by 2020 [18]. The use of MIS methods rose from 18.9% in 2002 to 98.6% in 2020 [18]. Also, among the MIS techniques, the application of laparotomy decreased from 62% to 29%, while the adoption of robotic technology rose from 26% to 61% [17]. In the USA, MIS is used for 65% of hysterectomy procedures, with robotic assistance for up to 43% of cases [19]. Given the widespread use and positive clinical outcomes of robotic methods, the present study was conducted to assess the short-term clinical outcomes of RH in a real-world setting in the Indian population.

## Materials and methods

This retrospective, real-world evidence study was conducted at the Department of Gynecology and Obstetrics at a tertiary care hospital. A retrospective chart review was performed for consecutive patients who underwent benign hysterectomy procedures via the robotic-assisted approach using the Da Vinci Si Surgical System between December 2021 and July 2024. Data collection was restricted to patients who had complete data on baseline characteristics and intra-operative and post-operative outcomes. The study was conducted according to the ethical principles specified in the latest edition of the Helsinki Declaration and the applicable guidelines for good clinical practice. The ethical approval for the study was obtained from the Zydus Hospital Ethics Committee.

Deidentified data for the pre-operative variables such as age, body mass index (BMI), and comorbidities were collected from the patient’s medical records. The intra-operative data including operating time, estimated blood loss, ambulation, length of hospital stay, and complications related to the procedure was also collected. Post-operative complications data for up to one week after surgery were also collected. All surgeries were performed by a single experienced surgeon.

Surgical technique

After the anesthesia was administered, the patient was placed in a semi-lithotomy position, and the surgical field was painted and draped. Foley's catheterization was a routine procedure for all our robotic cases. A colpotomizer-equipped vaginal manipulator was put in. Most of the cases in this study involved a three-arm approach. A supraumbilical 8-mm camera port and two accessory ports (8mm) 8-10 cm on either side of the main port were used. A 5-mm assistant port was placed on the left side between the camera port and the left accessory port, and above 5 cm, creating an equilateral triangle. To ensure proper visualization of the uterus and adnexa, a head-low position was provided. The robot was docked, and the targeting was done using fenestrated bipolar forceps in the left arm and monopolar curve scissors (hot shears) in the right arm. In most of our cases, an assistant's help was needed for bowel displacement, uterine manipulation, and suction irrigation. A mega needle driver or suture cut needle driver was used for vault sutures using V lock 1/0. Following surgery, the vagina was inspected for bleeding and the suture line was examined. The color and output of urine were recorded, and Monocryl 5/0 or surgical clips were used to make the port incision.

The data were recorded on a pre-designed form. Categorical variables were summarized using frequency and percentages. Continuous data were summarized using the arithmetic mean and standard deviation (SD). The data analysis was conducted using Stata statistical software Stata IC 13.1 (StataCorp LLC, College Station, Texas, USA).

## Results

The study included a total of 113 patients. The mean age and BMI of the study population were 45.39 ± 9.04 years and 29.07 ± 4.69 kg/m^2^, respectively. The main comorbidities were hypertension and hypothyroidism, both of which were present in 24 (21.24%) patients, and diabetes, which was present in 15 (13.27%) patients. Table 1 shows the descriptive characteristics of the pre-operative variables.

**Table 1:** Descriptive characteristics of pre-operative variables BMI, body mass index; SD, standard deviation

Variable	N=113
Age, mean ± SD, year	45.39 ± 9.04
BMI, mean ± SD, kg/m^2^	29.07 ± 4.69
Comorbidities, n (%)	
Hypertension	24 (21.24)
Hypothyroidism	24 (21.24)
Diabetes	15 (13.27)
Dyslipidemia	4 (3.54)
Asthma	2 (1.77
Chronic kidney disease	2 (1.77)
Heavy periods	2 (1.77)
Migraine	2 (1.77)
Severe dysmenorrhea	2 (1.77)
Abdominal pain	1 (0.88)
Anxiety	1 (0.88)
Constipation	1 (0.88)
Depression	1 (0.88)
Endometrial tuberculosis	1 (0.88)
Fever	1 (0.88)
Heart disease	1 (0.88)
Heavy bleeding with clots	1 (0.88)
Hyperthyroidism	1 (0.88)
Intramural fibroid	1 (0.88)
Left ear septum	1 (0.88)
Mild cystocele	1 (0.88)
Obstructive sleep apnea	1 (0.88)
Rheumatic arthritis	1 (0.88)
White discharge	1 (0.88)

The intra-operative and post-operative outcomes of the study population are given in Table 2. 

**Table 2:** Intra-operative and post-operative outcomes of the study population SD, standard deviation

Intra-operative and post-operative outcomes	N=113
Operative time, mean ± SD, min	178.41 ± 18.65
Estimated blood loss, mean ± SD, ml	23.85 ± 5.84
Ambulation, mean ± SD, hours	21.22 ± 3.10
Length of hospital stay, mean ± SD, days	2.86 ± 0.42
Post-operative complications, n (%)	
Urinary leakage	2 (1.77)
Intestinal obstruction due to ileal adhesion	1 (0.88)

The mean operating room time, estimated blood loss, and length of hospital stay were 178.41 ± 18.65 minutes, 23.85 ± 5.84 mL, and 2.86 ± 0.42 days, respectively. The mean ambulation time was 21.22 ± 3.10 hours. There were no intra-operative complications, whereas two (1.77%) patients had urinary leakage and one (0.88%) patient had intestinal obstruction due to ileal adhesion post-operatively. Urinary leakage may be due to damage to the pelvic floor or the nerve supply to the pelvic area.

## Discussion

Minimally invasive gynecologic surgery (MIGS) has transformed the science of gynecologic treatments in the past 20 years [9]. The guidelines for Enhanced Recovery After Surgery (ERAS) support the use of the MIS method as a tool to improve recovery after surgery [20]. The use of MIGS has increased due to the development and validation of robotic surgery, which has improved both surgical and patient outcomes [21]. Robotic surgery is a significant step forward in improving patient care by allowing for precise manipulations using stereoscopic vision, enlarged vision, and multi-joint functions [22,23].

The age range of our study population was in line with those of previous studies in India and other countries [24-26]. A single-centric study, which evaluated the real-time data of robotic surgery using the Da Vinci Si system for benign gynecological conditions in India over a decade, showed that the mean age of participants was 46.12 ± 6.77 years [24]. Another recent study conducted across five tertiary care hospitals in India reported that the mean age of benign cases was 40.84 ± 10.51 years [1]. The use of a robotic platform has been proven to provide better post-operative outcomes for patients undergoing benign hysterectomy when vaginal or laparoscopic approaches are not possible, especially for patients with high BMI [27]. Furthermore, research has shown that robotic surgery for benign hysterectomy is a viable and safe procedure, even in complex cases such as those with elevated BMI [17]. The mean BMI of patients in our study was 29.07 kg/m^2^, which was in line with a previous study from India that used RH for patients with a mean BMI of 28.40 kg/m^2^ [1]. A decade-long study on robotic surgery for benign gynecological conditions revealed that the average BMI was 28.89 kg/m^2^ [24]. Additionally, a different Indian study revealed that the mean BMI for benign pathology in the RH group ranged from 29.32 to 30.2 kg/m^2^ [24,25].

The robotic approach has been extensively researched and has the potential to provide perioperative benefits of MIS to patients who were previously only suitable for an open approach [19]. In our study, the total operating room time of the robotic group was considerably longer (178.41 ± 18.65 minutes). A previous study that compared the outcome of RH with LH for large uteri in Indian women also showed a longer operative time (131.0 minutes) [25]. Similarly, a meta-analysis also revealed that the robotic surgery group had a mean presurgical time (including port placement and docking time) of 15.56 minutes (range: 3-30 minutes), a mean console time of 83.21 minutes (range: 25-180 minutes), and a mean operative time of 136.6 minutes (range: 60-294 minutes) [11]. Furthermore, another study conducted in India on benign pathologies also revealed a longer mean total operative time of 127.37 ± 110.67 minutes for robotic myomectomy [28]. The longer operating times in robotic procedures may be caused by the time needed to set up, position, dock, and undock the robotic system before surgery, as per different studies [29-32]. Additional studies have shown that both the patient (such as older age, higher BMI, increased uterine weight, and adhesions) and the surgeon (technique, and expertise) can affect the length of the operating time [9,33,34]. Furthermore, research has demonstrated that robot docking durations are longer in the early learning phase and much shorter as the operating experience increases [35]. In a single-centric study in India, real-time data from a decade of robotic surgery for benign gynecological disorders was analyzed. The study concluded that docking time, console time, and operating time underwent significant and consistent improvements over time [24].

This study found that the RH group had a significantly lower estimated blood loss (23.85 ± 5.84 ml). A study that evaluated a single-site robotic hysterectomy for benign gynecologic pathology reported a mean blood loss of 43.68 mL (range: 15-300 mL) [11]. Furthermore, another study also found that the robotic group's mean estimated blood loss was significantly lower (50 ml, p<0.001) than the LH approach [15]. In addition, an Indian study discovered a lower decrease in hemoglobin levels in the RH group [25]. Improved vascular management by the robot could be attributed to its enhanced 3D visualization, instrumental dexterity, and precise resection, all of which contribute to decreased blood loss [36-41].

The robotic group in this study had a much shorter hospital stay (2.86 ± 0.42 days). These findings are in line with a retrospective analysis that showed a shorter mean length of stay of 2.07 days for benign cases [1]. Furthermore, a single-centric study in India indicated that 86.6% of patients stayed for less than 24 hours, 13.4% for more than 24 hours, and none for more than 72 hours [24]. A systematic review that evaluated single-site robotic hysterectomy for benign gynecologic pathology also showed that the median duration of hospital stay was 1.71 days (range: 0.96-3.5 days) [11].

Three (2.56%) out of the 113 patients in this study had post-operative complications, which indicates a reduced likelihood of post-operative complications among RH patients. Damage to the pelvic nerve supply and ileal adhesion following surgery could be the cause of urinary leakage and intestinal obstruction in our study population. These findings are in line with a systematic review that assessed a single-site robotic hysterectomy for benign gynecologic pathology and found that only three (1.4%) patients of the total experienced a post-operative complication [11]. Additionally, it can be stated that the observed post-operative complications (urinary leakage and intestinal obstruction caused by ileal adhesion) were minor. A notable decrease in post-operative complications can be attributed to the combination of robotic technology, surgical expertise, and the surgeon's ability to perform limited-access gynecological surgery [24].

Strengths and limitations

Limitations might be argued to include the retrospective design and a relatively shorter follow-up time. The study's generalizability may be limited since it is based on a single surgeon's experience at one hospital. The outcomes may reflect the surgeon's skill level rather than generalizable findings. Moreover, the study does not have a comparator arm, making it difficult to determine if the improvement in outcomes is solely due to RH. Two of the study's strengths are its quality data and large sample size. A prospective study on the Indian population seems warranted for evaluating the long-term outcomes of robotic surgery for benign hysterectomy.

## Conclusions

This study is a single-centered investigation into the use of RH in an Indian environment for patients with benign gynecological conditions. The study suggests that RH may have a potential advantage in terms of perioperative and post-operative outcomes. Moreover, reductions in intra-operative and post-operative complications could translate to reductions in the overall economic burden of treatment, with shorter hospitalization and concurrent improvements in quality of life. In conclusion, the findings of the study are preliminary and are restricted to a single-center context. Further research is suggested to validate the study's findings.
